# Transverse Myelitis: An Adverse Reaction to Abatacept

**DOI:** 10.7759/cureus.59201

**Published:** 2024-04-28

**Authors:** Adeniyi A Adelakun, Ahmad W Haddad, Noreen Mirza, Marcia Dover, Steven Golombek

**Affiliations:** 1 Internal Medicine, Saint Mary's General Hospital, Passaic, USA; 2 Internal Medicine, Saint Michael's Medical Center, Newark, USA; 3 Neurology, Saint Clare's Denville Hospital, Denville, USA; 4 Rheumatology, Saint Clare's Denville Hospital, Denville, USA

**Keywords:** rheumatoid arthritis, dmard, immune-related adverse events (iraes), abatacept, biologics

## Abstract

Immunotherapies are powerful disease-modifying agents in treating autoimmune diseases like rheumatoid arthritis (RA). However, their unique mechanisms of action confer a broad spectrum of immune-related adverse events (irAEs), which tend to be rare but complex, with significant risk for morbidity and mortality. We report a case of transverse myelitis in a patient with RA whose joint disease had been well-controlled with long-term intravenous abatacept. Suspicion of an unusual irAE in this elderly patient, whose neurologic symptomatology was gradual and protracted, prompted the discontinuation of abatacept and the rapid initiation of corticosteroid therapy. These interventions yielded a favorable clinical outcome for the patient. We must draw clinicians' attention to this rare but potentially consequential adverse drug reaction.

## Introduction

The pathogenesis of autoimmune joint diseases such as rheumatoid arthritis (RA), polyarticular juvenile idiopathic arthritis (pJIA), and psoriatic arthritis (PsA) involves the activation of T lymphocytes found in the synovium of affected joints [1]. Cellular activities, cytokine mediators, and products of T-lymphocyte activation play critical roles in the initiation, aggravation, and perpetuation of joint tissue damage in these conditions. Hence, therapies that inhibit T-lymphocyte activity have become pivotal in modifying the courses of these diseases [1,2]. The development and subsequent FDA approval of abatacept in 2005 expanded the therapeutic frontier regarding RA [3]. Extensive clinical data have accumulated over the years confirming the effectiveness of abatacept in RA [4-6], and its clinical success is further underscored by a recent clinical trial demonstrating its benefits in the prevention of RA in genetically high-risk subpopulations [7].

Generally, side effects and adverse reactions not recognized in trial phases I-III become apparent in post-marketing experience. We report the case of a 78-year-old Caucasian female who developed MRI-proven transverse myelitis in whom neurological symptoms ceased to progress following the discontinuation of abatacept and the initiation of an alternative disease-modifying antirheumatic drug (DMARD).

## Case presentation

A 78-year-old female with a history of well-controlled RA diagnosed at age 53, generalized anxiety disorder, dyslipidemia, and hypothyroidism presented with a six-month history of urinary retention. She had been on abatacept infusions monthly for eight years. Her presentation of progressive urinary retention was associated with bilateral lower extremity weakness (left greater than right), lower extremity numbness, and diminished perianal and urethral sensations. Her medications included alirocumab, levothyroxine, ezetimibe, and alprazolam.

Vital signs on admission include a temperature of 97.7 degrees Fahrenheit, a blood pressure of 140/67 mmHg, a heart rate of 56 beats per minute, a respiratory rate of 18 cycles per minute, and an oxygen saturation of 94% on room air. She was a well-oriented lady with a normal muscle build on physical examination. Neurological examination included intact cranial nerves. There was a sensory deficit at the L2 dermatomal region and reduced bilateral motor power in the lower limbs. Deep tendon reflexes in both lower extremities were normal.

Initial laboratories include a normal comprehensive metabolic panel, complete blood count, and Lyme serology. The patient underwent a lumbar puncture and cerebrospinal fluid (CSF) examination, which showed clear and colorless fluid. The sample was acellular with elevated protein. The oligoclonal band was unremarkable, and CSF, white blood cell (WBC), and red blood cell (RBC) were normal (Table 1).

**Table 1:** Analysis of CSF studies performed during the presentation. CSF: cerebrospinal fluid; WBC: white blood cell; RBC: red blood cell

Study	Value	Normal range
CSF glucose	40 mg/dL	40-80 mg/dL
CSF protein	105.8 mg/dL	15-45 mg/dL
CSF WBC	1.0/mm^3^	0.0-5.0/mm^3^
CSF RBC	4.0/mm^3^	0.0-5.0/mm^3^
CSF cytology	Acellular	-
Oligoclonal band	0	Less than 4

**Figure 1:**
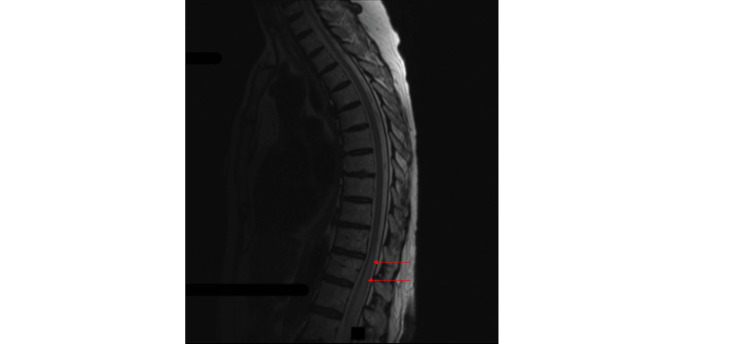
Non-contrast MRI (sagittal plane, T2) of the thoracic and lumbar spine, done at admission. The study demonstrates myelitis of the conus medullaris.

**Figure 2:**
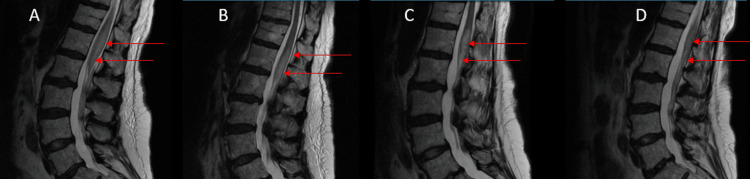
Non-contrast MRI of the lumbar spine. (A) At admission. (B) At discharge, nine days after admission. (C) Four months after discharge. (D) Six months after discharge. Serial images taken from presentation up to four months after admission show persistent lumbar spine signal changes. The MRI done in the sixth month shows improvement in the lesion after the discontinuation of abatacept.

The MRI scans of the thoracic and lumbar spine at admission demonstrated expansion of the conus medullaris and edema of the cord extending from T11 through the tip of the conus medullaris (Figure 1 and Figure 2A-2C). She received parenteral methylprednisolone 250 mg six hourly, followed by prednisolone 20 mg daily for 10 days after discharge, before reducing the dose to 10 mg daily. As the etiology of the myelitis was uncertain, she continued on abatacept.

The patient continued to have urinary incontinence and lower extremity weakness when evaluated five months later. A repeat MRI of the spine showed the persistence of the myelitis (Figure 2C). At this point, abatacept was discontinued, and the patient was started on rituximab.

Seven months post-discharge, she reported improved urinary incontinence but had persistent, minimally decreased sensation in the left leg. Clinical examination revealed preserved strength at the psoas, quadriceps, hamstrings, dorsiflexion, and ankle plantar flexion. There was decreased sensation to light touch on the toes. Pinprick sensation was preserved bilaterally in both lower extremities. Babinski sign was absent, reflexes were somewhat diminished, and gait was antalgic. MRI scan of the lumbar spine showed improvement in the cord signal intensities (Figure 2D). 

## Discussion

Abatacept has been extensively used to treat RA by interfering with T-cell immune activity. It functions as a fusion protein composed of the Fc region of IgG1 fused to the extracellular domain of CTLA-4 [4]. Its binding to CD80 and CD86 molecules on antigen-presenting cells inhibits T-cell activation [4,8]. Although preventing T-cell activation by interfering with signaling via CD28 still represents its main mechanism of action, abatacept also acts on additional immune cell populations such as regulatory T cells, monocytes/macrophages, osteoclasts, and B cells [8]. 

Abatacept is indicated in managing adult RA, pJIA, PsA, and prophylaxis for acute graft-versus-host disease. While it has been proven effective in managing conditions whose etiology is based on T-cell activation, like RA and autoimmune myocarditis [9], these theories did not turn out to be true when abatacept was trialed on patients with relapsing-remitting multiple sclerosis (RRMS) with activated T-cell-based pathogenesis [10]. 

Secondary transverse myelitis can be related to other immune-related conditions like multiple sclerosis (MS). Patients with MS are usually, unlike the case we presented, between the ages of 20 and 40. However, white ethnicity is predisposed; in our case, CSF studies and the oligoclonal band were not suggestive of MS. Neuromyelitis optica spectrum disorder (NMOSD), which may present with similar longitudinal extensive spinal cord lesion spanning three or greater vertebral segment, is another secondary cause of transverse myelitis. Still, patients are usually Asian and black, younger in age, specifically 40-60 years old, with usually more than one core clinical characteristic like bilateral optic neuritis; NMOSD is not related to autoimmune disease [11]. Myelin oligodendrocyte glycoprotein antibody-associated disease (MOGAD) is another condition that may cause transverse myelitis. Still, it's associated with relapsing and bilateral optic neuritis, brainstorm encephalitis, and acute disseminated encephalomyelitis (ADEM); MOGAD is more common in children than adults; 60% have CSF pleocytosis and can mimic the NMOSD syndrome seen in patients with the anti-AQP4 antibody [12,13]. In the case we present, there is a persistence of clinical symptoms and MRI changes that lasted for months until abatacept was discontinued, despite treatment with high-dose steroids. Rituximab, a replacement for abatacept for the treatment of RA, could be used as a maintenance treatment in NMOSD; however, evidence is scarce at this moment.

The exact underlying pathogenesis of irAEs has yet to be fully understood. It is proposed that it may be related to expanding the T-cell repertoire, some of which are autoreactive. In addition, the effect on B-cell responses may lead to the induction of autoantibody production [14]. The experience from the use of tumor necrosis factor (TNF) inhibitors, which are highly effective in treating autoimmune rheumatic diseases like RA, is that they increase the risk of developing inflammatory demyelinating CNS events [15]. While well-reported with TNF inhibitors, transverse myelitis and other neurologic complications, like CNS demyelination from abatacept, are unreported [14,16]. 

The patient presented here developed transverse myelitis eight years into treatment with abatacept [17,18]. While continuing on abatacept, MRI and clinical findings of transverse myelitis persisted. It was not until abatacept was discontinued five months later that the MRI and clinical findings improved, suggesting a causal relationship.

## Conclusions

While immunotherapies such as abatacept are effective in treating autoimmune diseases, including RA, they can uncommonly result in severe adverse events related to the immune system (irAEs). This case of transverse myelitis in a patient with RA on abatacept highlights the significance of identifying and quickly treating such irAEs. A favorable clinical outcome was achieved by stopping abatacept and starting corticosteroid therapy before switching to another biologic DMARD.
